# A linear model for predicting performance of short-read aligners using genome complexity

**DOI:** 10.1186/1471-2105-16-S15-P17

**Published:** 2015-10-23

**Authors:** Quang Tran, Shanshan Gao, Nam S Vo, Vinhthuy Phan

**Affiliations:** 1 Department of Computer Science, University of Memphis, TN 38152, USA

## Background

The effectiveness and accuracy of aligning short reads to genomes have an important impact on many applications that rely on next-generation sequencing data. The computational requirements and material cost for aligning large-scale short reads to genomes is also expensive. To prevent wasted time and resources for aligning short reads, we investigated the different measures of genome complexity[1] that correlated best to the performance of alignment to propose a linear model for each aligning method[2].

## Materials and methods

We demonstrated that repeats in genomic DNA could affect greatly the performance of short-read aligners. Exploring several different measures of genome complexity, we showed that there was a high correlation between our proposed-measure of genome complexity and, respectively, alignment accuracy and chromosomal coverage. The result was validated using 9 state-of-the-art aligners (Bowtie2[3], BWA-SW[4], Cushaw2, Masai[5], mrFast, SeqAlto, SHRiMP2[6], Smalt, and SOAP2[7]) and two different data sets. The first dataset, which consists of 100 genomic sequences including bacteria, plants and eukaryotes, was used to correlate complexity and alignment accuracy. The second dataset, which consisted of all 24 chromosomes of human, 20 chromosomes of soybean, and 10 chromosomes of corn, was used to correlate complexity and chromosomal coverage. High correlation between alignment performance and complexity enabled us to build linear regression models that could predict alignment accuracy and chromosomal coverage.

## Results

We demonstrated the utility of this method by showing how to use linear models to predict accuracy of aligners simply based on the complexity of genomes without using any reads for alignment. This can potentially help reduce experimental costs. Further, we showed how to use linear models to predict chromosomal coverage of genomes based on the expected coverage. This ability can also help reduce experimental costs as it allows researchers to predict how much a given number of reads will effectively cover chromosomes of interest. A visualization of genome complexity along chromosomes will also help to visually identify chromosomal regions that are potentially difficult to be covered by reads.

## Availability

Software to compute measures of genome complexity is available at https://github.com/vtphan/shortread-alignment-prediction

## References

[B1] Lynch M Conery JS The origins of genome complexity Science 2003 302 1401 1404 10.1126/science.1089370 14631042

[B2] Kärkkäinen J Sanders P Burkhardt S Linear work suffix array construction Journal of the ACM (JACM) 2006 53 918 936 10.1145/1217856.1217858

[B3] Langmead B Salzberg SL Fast gapped-read alignment with Bowtie 2 Nature Methods 2012 9 357 359 10.1038/nmeth.1923 22388286 PMC3322381

[B4] Li H Durbin R Fast and accurate long-read alignment with Burrows–Wheeler transform Bioinformatics 2010 26 589 595 10.1093/bioinformatics/btp698 20080505 PMC2828108

[B5] Siragusa E Weese D Reinert K Fast and accurate read mapping with approximate seeds and multiple backtracking Nucleic Acids Research 2013 41 e78 e78 10.1093/nar/gkt005 23358824 PMC3627565

[B6] David M SHRiMP2: sensitive yet practical short read mapping Bioinformatics 2011 27 1011 1012 10.1093/bioinformatics/btr046 21278192

[B7] Li R SOAP: short oligonucleotide alignment program Bioinformatics 2008 24 713 714 10.1093/bioinformatics/btn025 18227114

